# The role of long non-coding RNA in Crohn's disease

**DOI:** 10.1016/j.heliyon.2024.e32606

**Published:** 2024-06-06

**Authors:** Guo Chen, Heng Deng, Ming Li, Xiaoli Fang, Chunrong He, Yingzi Shu, Feifei Wang

**Affiliations:** a Department of Internal Medicine, Anhui Hospital affiliated Shanghai Shuguang Hospital, Hefei, Anhui, China; b Department of Anorectal Surgery, the Second Affiliated Hospital of Anhui University of Chinese Medicine, Hefei, Anhui, China; c Department of Anorectal Surgery, the First Affiliated Hospital of Anhui University of Chinese Medicine, Hefei, Anhui, China; d Hefei Haiheng Health Service Center, Hefei, Anhui, China

**Keywords:** Long non-coding RNA, Crohn's disease, Biomarkers

## Abstract

Emerging evidence has illuminated the pivotal role of long noncoding RNAs (lncRNAs) in orchestrating immunological functions and autoimmune responses. In the context of Crohn's disease (CD), an array of novel lncRNAs has been identified in the plasma and intestinal tissues of afflicted individuals, suggesting a dualistic influence on the disease progression, either exacerbating or mitigating its course. Current research has demonstrated the involvement of lncRNAs in competitive endogenous RNA, the inflammation process, epithelial barrier function, gut microbiota imbalance, and epigenetic regulation. This review aims to encapsulate the current knowledge on the lncRNA contribution to CD and underscore potential avenues for future research. LncRNAs are increasingly recognized as significant biomarkers and potential therapeutic targets, holding a key position in the pathogenesis of CD. Furthermore, the unique attributes of circulating lncRNAs, such as minimal side effects, combinational therapy potential, and personalized medicine, render them as promising therapeutic tools for individual health management in CD.

## 1 Introduction

Crohn's disease (CD) is a chronic inflammatory bowel disease that can affect any segment of the gastrointestinal tract, from the oral cavity to the anal canal [1]. It is more commonly observed in developed countries, particularly in urban and industrialized regions. As developing countries adopt Western lifestyles and dietary habits, the incidence of CD is on the rise. Incidence is more common in young adults but can affect individuals of any age, with a rising prevalence [2]. A distinctive pathological aspect of CD is that inflammation often extends deep into the layers of the affected bowel tissue, potentially resulting in strictures, fistulas, abscesses, and malnutrition [3]. Clinically, CD is typified by alternating periods of exacerbation and remission, manifesting symptoms such as abdominal pain, diarrhea, weight loss, and fatigue. These symptoms do not exhibit a distinct preference for gender or ethnicity [[4], [5]]. The course of CD is marked by its variability and unpredictability, and it is associated with an increased long-term risk for colorectal cancer [6]. The etiology of CD remains elusive, but it is believed to involve a complex interplay of environmental [7], genetic [8], and immune factors [9]. Immune dysregulation, inflammatory response, and genetic susceptibility triggered by unknown antigens, such as smoking [10], diet [11], and infectious agents [12]. These factors pose significant challenges for diagnostic evaluation and treatment strategies.

Long non-coding RNAs (lncRNAs) characterized by their length exceeding 200 nucleotides and their lack of protein-coding capacity, represent a distinct class of RNA molecules. These molecules are recognized for their role as diagnostic markers and therapeutic targets in various diseases, including CD [13]. The Dysregulation of lncRNA-mediated processes contributes to the development of CD, which includes inflammatory response, immune regulation, intestinal tissue damage repair [14]. LncRNAs exhibit a more extensive range of regulatory mechanisms in CD compared to other non-coding RNAs, including autophagy regulation, DNA methylation, and histone modification. Moreover, lncRNAs can exert regulatory effects on other non-coding RNAs [15]. As diagnostic markers, lncRNAs offer several advantages, including their stability and conservation across different tissues and species, their distinct expression patterns in different disease states, and their non-invasive detectability in body fluids such as blood, saliva, and urine [16]. Additionally, they hold promising therapeutic potential by modulating the activity of multiple genes through the regulation of lncRNA expression. Conversely, novel therapeutic options involving the inhibition of lncRNAs, such as molecule inhibitors and antisense oligonucleotides, are currently under investigation. Recent studies have highlighted the association between abnormal lncRNA expression and dysregulation with the pathogenesis and progression of CD. This review aims to critically evaluate and synthesize the current literature to gain an updated understanding of the regulatory role of lncRNAs in the pathogenesis of CD. A comprehensive search was conducted using the PubMed and Scopus databases, along with relevant keywords such as "long non-coding RNA," "Crohn's disease," or "inflammatory bowel disease." The search results were meticulously reviewed, and relevant research articles were selected based on their alignment with the objectives of this study. The extracted data were then meticulously analyzed, categorized, and integrated to delineate the roles of lncRNAs in both promoting and inhibiting CD, as well as to explore their potential mechanisms of action.

### 1.1 1. LncRNAs contribute to the biology of CD

Differential expression of lncRNAs has been observed in inflamed intestinal tissues of CD patients compared to healthy individuals. These lncRNAs are involved in the regulation of key characteristics of CD, such as immune dysfunction and uncontrolled inflammation. In the subsequent sections, we will discuss several lncRNAs that promote these pathogenic characteristics, with a specific emphasis on those that are associated with a poor prognosis in CD patients (summarized in Table 1).

**Table 1:** Known lncRNAs dysregulated in CD.

LncRNA	Sample source	Assay method	Location	Effect on CD	Ref.
MSC-AS1	Human and Rats colonic tissue	qRT-PCR	chr8:72,279,244-72,491,399	Activate EMT	[14]
H19	Human blood	qRT-PCR	chr11:1,995,176–1997835 (−)	Promote inflammation	[19 20]
MALAT1	Human and mice intestinal mucosal tissue	Microarray/qRT-PCR	chr11:65497688–65506516 (+)	Impaired mucosal barrier	[22 23]
LUCAT1	Human blood	qRT-PCR	chr5:91054834-91314547 (−)	Promote inflammation	[25]
LINC01272	Human Colonic and ileal tissue	qRT-PCR/Microarray	Chr20:NC_000020.11	Activate EMT	[[26], [27], [28]]
DQ786243	Human blood	qRT-PCR	chr1:155234457-155239960	Generate regulatory T lymphocytes	[29]
MEG3	Human colonic tissue	qRT-PCR	Chr14:NC_000014.9	Promoted TNF-α	[31]
THRIL	Human blood	qRT-PCR	Chr12:NC_000012.12	Promote inflammation	[33]
CNN3-206	Human colonic tissue	qRT-PCR	Chr1p21.3	Upregulate Caspase10	[34]
XLOC_000261	Human terminal ileal samples	Microarray	chr7:155,799,529–155,812,871	Promote differention of Th17 cells	[35]
XLOC_000639	Human terminal ileal samples	Microarray	Chr4:rs4692386	Promote differention Th1 and Th17 cells	[35]
XLOC_000014	Human terminal ileal samples	Microarray	Chr1:rs7547569	Promote differention Th1 and Th17 cells	[35]
ITSN1-2	Human intestinal mucosa samples	Microarray	Chr21:NC_000021.9	Promote CD4^+^ T cell activation and Th1/Th17 cell differentiation	[36]
LINC00641	Human blood	qRT-PCR	chr14:15397491–15402915	Promoted TNF-α	[39]
ANRIL	Human intestinal mucosa samples/blood	qRT-PCR	Chr. 9p21	Reduce TNF-α, IL-17	[42]
HIF1A-AS1	Human colonic tissue	qRT-PCR/Microarray	Chr14:NC_000014.9	Alter gut microbes	[45]
HIF1A-AS2	Human colonic tissue	Microarray	Chr14:NC_000014.9	Promote Flagellin-mediated anti-inflammatory affects	[47]
Lnc78583	Human Colonic tissue	qRT-PCR/Microarray	Chr.17: 48,752,528‍‒‍48,755,853	Reduce LPS-induced colorectal cell inflammation	[48]
LHFPL3-AS2	Human ileum tissue	qRT-PCR	Chr7:NC_000007.14	Promote epithelial polarity and proliferation	[49]
LOC102550026	Rats colonic tissue	qRT-PCR/Microarray	Chr5:NC_005104.4	Reduce TNFα, IL-1β, and IL-6	[50]

LncRNA MSC-AS1 from intestinal tissues promotes Fibrotic Pathology.

LncRNA MSC-AS1 has been found to be highly expressed in pancreatic cancer, hepatocellular carcinoma and glioma [17]. In the intestinal tissue of CD mice induced by 2,4,6-trinitrobenzenesulfonic acid, MSC-AS1 was not only highly expressed but also induced intestinal fibrosis by promoting epithelial-mesenchymal transition (EMT) [14].

### 1.2 H19 from blood exacerbates the severity of CD

LncRNA H19 is a single-stranded RNA molecule approximately 2.3 kb long in humans, located on human chromosome 11p15.5. Initially identified as an oncogene in various tumor patients [18]. H19 has recently been found to be upregulated in CD patients. Increased serum levels of H19 showed a statistically significant difference between CD patients and healthy controls. Moreover, high expression of H19 was closely associated with increased disease severity and the need for surgical intervention [19]. H19 has also demonstrated excellent diagnostic ability in distinguishing active CD from CD in remission [20].

Metastasis-associated lung adenocarcinoma transcript 1 (MALAT1) from intestinal tissues intensifies the activity of CD.

LncRNA MALAT1 initially identified in non-small cell lung cancer, is a lncRNA transcribed from the nuclear-enriched transcript 2 (NEAT2) gene. This 7.5 kb transcript has been implicated in various diseases, particularly in cancers [21] and autoimmune disorders. Notably, MALAT1 is highly expressed in the gastrointestinal tract and is associated with the pathogenesis of CD. Its dysregulation is linked to increased production of pro-inflammatory cytokines and disease severity in CD [22]. Furthermore, the expression of MALAT1 is closely linked to an increased risk of CD, higher disease activity, and worse clinical outcomes [23].

### 1.3 Lung cancer associated transcript 1 (LUCAT1) from blood promotes the recurrence of CD

LUCAT1, an inflammatory regulator, exhibits significantly increased expression in the intestinal tissues of CD patients compared to healthy individuals [24]. Elevated LUCAT1 expression is associated with disease severity and progression [25], and it plays a crucial role in transitioning patients with CD from the remission phase to the active phase, as observed in both clinical and endoscopic assessments. Moreover, lncRNA LUCAT1 shows a positive correlation with erythrocyte sedimentation rate, C-reactive protein, fecal calprotectin, and CD activity index.

LINC01272 from colonic and ileal tissue was positively correlated with mucosal injury.

LINC01272, a lncRNA molecule located on chromosome X, is transcribed into RNA but does not encode a protein. It has been found to be upregulated in various cancers, including lung cancer and colorectal cancer [26]. Furthermore, LINC01272 has been identified as upregulated in inflamed intestinal tissues of CD patients compared to normal tissues of healthy subjects undergoing colonoscopy [27]. Notably, the overexpression of LINC01272 positively correlates with more severe mucosal injury in CD biopsy tissue [28].

### 1.4 LncRNA DQ786243 from blood increases inflammation

DQ786243, prominently overexpressed in blood samples, has been identified as a potential biomarker for distinguishing between clinically active and inactive CD patients through Quantitative RT-PCR analysis [29]. This observation suggests that LncRNA DQ786243 may contribute to the pathogenesis of CD by influencing immune response and inflammation.

### 1.5 Maternally expressed gene 3 (MEG3) from colonic tissue increases anal abscess

MEG3, located on chromosome 14q32 and composed of two exons, is recognized for its tumor-suppressive properties, which are exerted through the regulation of proliferation, apoptosis, differentiation, and metabolism [30]. Notably, MEG3 expression levels are significantly elevated in the peripheral blood of CD patients compared to healthy individuals [31], indicating its potential involvement in exacerbating the inflammatory response associated with anal abscess, a common complication of CD.

TNF-α and heterogeneous nuclear ribonucleoprotein L (THRIL) from blood elevate intestinal complications.

THRIL, also known as TNF-α related immunoregulatory lncRNA [32], is transcribed from the LOC105378488 gene located on human chromosome 3. It is primarily expressed in immune cells and involved in the regulation of genes associated with the immune response and inflammation. Compared to controls, THRIL is significantly up-regulated in CD patients, particularly in patients with intestinal perforation and colonic stricture [33].

### 1.6 LncRNA-CNN3-206 from colonic tissue contributes to the pathogenesis

LncRNA-CNN3-206, a transcript originating from the CNN3 gene on human chromosome 11, is highly conserved across species, suggesting its functional significance. It is believed that lncRNA-CNN3-206 may contribute to the pathogenesis of CD by promoting the expression of genes involved in the immune response and inflammation in the gastrointestinal tract [34].

### 1.7 XLOC_000261 from terminal ileal tissue enhance the aggregation of inflammatory cells

In the terminal ileal tissue, quantitative polymerase chain reaction revealed a significant overexpression of lincRNA XLOC_000261, XLOC_000639, and XLOC_000014 in CD4^+^ cells from CD patients when compared to healthy controls [35]. This observation diverges from the previously noted abnormal expression of lncRNA, which was limited to biopsy tissues or large tissues.

### 1.8 ITSN1-2 from intestinal mucosa tissue facilitates the release of inflammatory cytokines

Regarding ITSN1-2, this lncRNA expressed intestinal mucosa tissue was found to be elevated in both ulcerative colitis and CD patients. The increase in expression positively correlates with markers such as CRP, ESR, CDAI score, TNF-α, IL-17, IL-1β, and IL-18. Interestingly, the administration of infliximab to CD patients has been observed to downregulate both plasmic and intestinal mucosa lnc-ITSN1-2 expression [36].

### 1.9 LINC00641 from blood is associated with TNF-α levels

LINC00641, situated on chr14:15397491–15402915, has been extensively reported in various cancers, including papillary thyroid [37], lung Adenocarcinoma, lung Squamous cell carcinoma, renal cell carcinoma, and colorectal cancer [38], where it plays contrasting biological roles. There is a single correlation study between LINC00641 and CD, indicating that LINC00641 in the blood of CD patients is significantly elevated and positively correlated with TNF-α levels [39].

## 2 LncRNAs inhibit to biological processes of CD

In contrast, lncRNAs also exert a onsiderable influence in suppressing multitude of biological processes pertinent to CD. A comprehensive understanding of the roles of lncRNAs play in the pathogenesis of CD can offer invaluable insights into innovative therapeutic strategies. LncRNAs possess the capability to downregulate the expression of pro-inflammatory genes, such as IL-1β, TNF-α, and IL-6. By inhibiting the activity of these inflammatory mediators, lncRNAs contribute to the mitigation of inflammation associated with CD.

Antisense noncoding RNA in the INK4 locus (ANRIL) from intestinal mucosa tissue and blood reduces disease activity.

A notable example of a LncRNA is the antisense noncoding RNA in the INK4 locus (ANRIL). ANRIL is a gene located on human chromosome 9p21 and is transcribed from the INK4B-ARF-INK4A gene cluster. It plays a crucial role in suppressing tumor growth by regulating the expression of nearby protein-coding genes involved in cell proliferation, senescence, apoptosis, and DNA repair [40]. The expression of ANRIL has been observed to be decreased in both CD patients [41] and active CD patients, as compared to healthy individuals and those in remission from CD. Furthermore, it has been negatively associated with inflammatory markers such as C-reactive protein, erythrocyte sedimentation rate, TNF-α, and IL-17 [42]. Similar findings have been observed in children with CD [43].

### 2.1 HNF1A-AS1 from colonic tissue inhibits the pathogenesis

HNF1A-AS1 is transcribed from the antisense strand of the HNF1A gene. It is also known as HNF1A upstream transcript 1 or HNF1A upstream transcription factor A. As a single-stranded RNA molecule, it can fold into secondary structures, enabling interactions with other molecules and proteins [44]. The reduction of HNF1A-AS1 in mucosal biopsies of patients with CD has been confirmed, compared to healthy subjects [45].

### 2.2 HIF1A-AS2 from colonic tissue mitigates inflammatory cytokines

HIF1A-AS2, also known as long intergenic non-protein coding RNA 2055 (LINC02055) or HOX transcript antisense RNA (HOTAIR), resides on chromosome 14q23.2 [46]. It is transcribed from the antisense strand of the HIF1A gene, encoding the hypoxia-inducible factor 1-alpha (HIF-1α) transcription factor. In CD patients, HIF1A-AS2 deregulates TNF-α, IL-1β, IL-6, and IL-12, and promotes flagellin-mediated anti-inflammatory effects [47].

### 2.3 Lnc78583 from colonic tissue inhibits inflammation

Lnc78583 is situated at chromosome 17 (chr17): 48,752,528‍‒‍48,755,853. It is significantly decreased in CD, along with highly expressed TNF-‍α and interleukin-6. There is a strong positive correlation between the anti-inflammatory factor IL-10 and lnc78583. Research has shown that the overexpression of lnc78583 inhibits lipopolysaccharide (LPS)‍-induced colorectal cell inflammation [48].

### 2.4 LHFPL3-AS2 from ileum tissue inhibits mucosal injury

LHFPL3-AS2 is reduced in CD patients compared to healthy subjects, with a more pronounced reduction in patients with severe mucosal injury. Studies have demonstrated that LHFPL3-AS2 knockdown cells lack polarity, epithelial morphogenesis, mitotic spindle formation, and proliferation [49].

### 2.5 LOC102550026 from colonic tissue is associated with disease remission

The expression level of LOC102550026 is markedly decreased in CD, but it can be increased after effective treatment [50]. Studies on the high expression of LOC102550026 in CD samples have been limited to animal studies.

## 3 Mechanisms of lncRNA involvement in CD

**Fig. 1:**
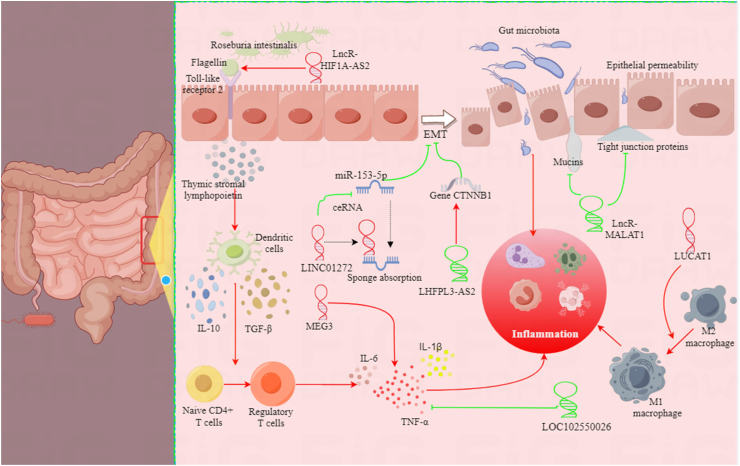
legend: An overview of the mechanism of lncRNAs in CD. Representative mechanism for lncRNA affects the CD process: LncRNAs LHFPL3-AS2 act as guides or scaffolds, recruiting transcription factors or chromatin modifiers to specific genomic loci through base pairing, affecting EMT. LncRNAs LINC01272 can also compete with microRNA-153-5p for binding to complementary target sites, thus inhibiting miRNA-mediated gene silencing. LncRNAs can also act as scaffolds for protein complexes, facilitating their assembly or modifying their activity, to generate recruitment of inflammatory cells. LncRNAs MEG3 and loc102550026 regulates immune response and inflammation by modulating the expression of pro-inflammatory cytokines, including TNF-α and IL-6, IL-1β. LncRNAs MALAT1 can regulate of epithelial barrier function by modulating tight junction proteins and mucins. HIF1A-AS2 can interact with roseburia intestinalis through toll-like receptor 5 incubated human monocyte-derived dendritic cells, promote the differentiation of anti-inflammatory T cells, thereby inhibiting the development of CD. LncRNAs LUCAT1 regulates the activation and polarization of macrophages, and promotes the inflammatory process.

Aberrant lncRNAs have been identified as pivotal regulators in pivotal biological processes associated with CD, including inflammation, immune response, and epithelial barrier function (Fig. 1).

### 3.1 Competitive endogenous RNA (ceRNA) hypothesis

It has been proposed that certain lncRNAs may function as molecular sponges for microRNAs, thereby modulating the expression of target mRNAs involved in the pathogenesis of CD. For example, lncRNACNN3-206 binds to miR-212, leading to a decrease in its effective level, which in turn may up-regulate the expression of Caspase10. LOC102550026 reduces miR-34c-5p through sponge adsorption, thereby inhibiting the reduction of human recombinant Protein Pck1 [50]. Lnc-ITSN1-2 acts as a sponge for miR-125a, promoting the progression of CD. Additionally, RNA-protein interactions have been observed, such as the interaction between lncRNAs ISR8 and Zinc finger proteins, which can modulate their functions and influence various cellular processes implicated in Crohn's disease [51].

### 3.2 Role of lncRNAs in immune response and inflammation

LncRNAs have been demonstrated to serve as regulators of immune response and inflammation in CD. They possess the capability to modulate the expression of pro-inflammatory cytokines, including TNF-α [52] and IL-6, IL-1β, and IL-12 [47]. LncRNAs such as LUCAT1 have been shown to regulate the activation and polarization of macrophages, which play a crucial role in the inflammatory process [24].

### 3.3 Epithelial barrier function and lncRNAs

The impairment of the intestinal epithelial barrier is a characteristic feature of CD [53]. Dysregulation of these lncRNAs can lead to barrier dysfunction, increased intestinal permeability, and subsequent immune activation [54]. One such lncRNA, MALAT1, has been implicated in the regulation of epithelial barrier function by modulating tight junction proteins and mucins [22]. The downregulation of LHFPL3-AS2 expression inhibits epithelial polarity and proliferation, directly leading to the translocation of intestinal bacteria across the mucosal barrier, triggering inflammation throughout the intestinal wall [49].

### 3.4 LncRNAs and gut microbiota dysbiosis

Gut microbiota dysbiosis is closely associated with the pathogenesis of CD [55]. Emerging evidence suggests that lncRNAs can regulate the composition and function of the gut microbiota [56]. For instance, through toll-like receptor 5 incubated human monocyte-derived dendritic cells, roseburia intestinalis promotes the differentiation of anti-inflammatory T cells, thereby inhibiting the development of CD [57]. Another lncRNA, HIF1A-AS2, can interact with roseburia intestinalis and influence the host-microbiota interaction in CD [47].

### 3.5 Epigenetic regulation

Aberrant expression of lncRNAs can influence DNA methylation patterns and histone modifications, resulting in altered gene expression profiles in CD [58]. Certain lncRNAs can interact with chromatin-modifying proteins and regulate the expression of key genes in CD.

## 4 Therapeutic potential of targeting lncRNAs

The dysregulation of lncRNAs in CD makes them promising therapeutic targets. By targeting lncRNAs, new therapeutic strategies for CD could be developed, potentially offering fewer side effects. Here are some therapeutic approaches that focus on targeting lncRNAs in CD: 1. Small interfering RNAs (siRNAs): These molecules can specifically target and degrade lncRNAs, thereby reducing their abnormal expression [59]. 2. Antisense oligonucleotides (ASOs): ASOs can bind to lncRNAs and prevent their interaction with other molecules, thereby modulating their function [60]. 3. CRISPR-Cas9: This gene editing tool can be utilized to target and modify specific lncRNA sequences [51]. 4. Small molecule inhibitors: These compounds have the ability to inhibit the activity of lncRNAs or their interactions with other molecules. By exploring these therapeutic approaches, it may be possible to harness the therapeutic potential of lncRNAs and develop more effective treatments for CD.

## 5 Discussion

Crohn's disease is a chronic inflammatory condition that primarily affects the gastrointestinal tract, manifesting as inflammation that can span various segments of the digestive system. The etiology of CD remains elusive, but it is widely accepted that a complex interplay of genetic susceptibility, environmental triggers, and aberrant immunological responses contributes to its onset. The immune system erroneously attacks the gut, leading to inflammation and structural damage. Symptomatology of CD is heterogeneous and can encompass a spectrum of presentations, with abdominal pain, diarrhea, unintended weight loss, fatigue, and bloody stools. The course of CD can fluctuate from relatively benign to profoundly disabling, with potential complications such as strictures, fistulas, and abscesses. The therapeutic approach to CD is multifaceted, aiming to quell inflammation, mitigate symptoms, preserve or restore quality of life, and forestall the onset of complications. Nonetheless, patients often encounter a treatment dilemma due to the diverse presentations of the disease, potential side effects, and limited treatment options. Current treatments for CD are not always effective and can have significant side effects. Pharmacological treatment options include aminosalicylates, corticosteroids, immunomodulators, and biologic agents. Additionally, surgical intervention, dietary adjustments, and lifestyle modifications, are also integral components of a comprehensive management strategy.

Individualized treatment plans should consider factors such as disease severity, location, complications, and patient preferences. The advent of targeted biologic therapies was anticipated to revolutionize the treatment of CD. LncRNAs are RNA molecules that do not encode proteins but play crucial roles in regulating genes. Targeting lncRNAs in CD offers several potential benefits: ①Specificity: By targeting lncRNAs, it is possible to regulate genes precisely without affecting protein-coding genes. ②Reduced side effects: Compared to existing treatments, therapies that target lncRNAs may have fewer adverse effects. ③Combination therapy: Targeting lncRNAs can be used in conjunction with current therapies to enhance their effectiveness. ④Personalized medicine: lncRNAs have the potential to serve as biomarkers for disease prognosis and treatment response. Targeted biologic therapies aim to specifically target lncRNAs associated with CD, providing more effective and personalized treatment options. In addition, the emerging role of lncRNAs has the potential to revolutionize the diagnostic landscape of CD. LncRNAs, as promising biomarkers, offer valuable insights into early detection, disease classification, and prognosis evaluation. Notably, these molecules can be detected in non-invasive samples such as blood, tissue, or stool, which significantly enhances the accessibility of diagnostic testing. Furthermore, integrating lncRNA expression data with other clinical indicators and biomarkers, such as inflammatory markers like TNF-α, can significantly refine the diagnostic precision and sensitivity for CD. This combined approach enables a more comprehensive evaluation of inflammation levels and disease progression, thereby facilitating tailored treatment strategies and improved patient outcomes.

However, further research is imperative to elucidate the functional roles of specific lncRNAs and to evaluate their therapeutic potential in both preclinical and clinical studies. There are several challenges and future directions in the study of the relationship between lncRNAs and CD. These include the identification of disease-specific lncRNAs and the comprehension of their functional roles. Additionally, there is a pressing need to develop efficient and safe delivery systems for lncRNA-targeting therapies, while also ensuring specificity and minimizing unintended consequences of targeting lncRNAs. Furthermore, further research and clinical trials are necessary to validate the efficacy and safety of lncRNA-targeting therapies.

In conclusion, the emerging role of lncRNAs in CD highlights their potential contribution to disease pathogenesis and progression. Gaining insights into the dysregulation and functional roles of specific lncRNAs can provide valuable understanding of the underlying mechanisms of CD and potentially lead to the development of novel diagnostic markers and therapeutic interventions.

## Ethics declarations

Review and/or approval by an ethics committee was not needed for this study because this is a review.

## Funding

This research was funded by Scientific Research Project of Colleges and Universities of Anhui Province (No. 2023AH050848); and Xinglin talent training program (No. 0500-48-30)

## Data availability statement

Data included in article/supp. material/referenced in article.

## CRediT authorship contribution statement

**Guo Chen:** Writing – original draft. **Heng Deng:** Data curation, Conceptualization. **Ming Li:** Resources, Formal analysis, Conceptualization. **Xiaoli Fang:** Resources. **Chunrong He:** Resources. **Yingzi Shu:** Data curation. **Feifei Wang:** Methodology.

## Declaration of competing Interest

None.
